# Depending on Epitope Profile of COVID-19 mRNA Vaccine Recipients: Are They More Efficient Against the Arising Viral Variants? An Opinion Article

**DOI:** 10.3389/fmed.2022.903876

**Published:** 2022-06-20

**Authors:** Nawal Abd El-Baky, Amro Abd Al Fattah Amara

**Affiliations:** Department of Protein Research, Genetic Engineering and Biotechnology Research Institute (GEBRI), City of Scientific Research and Technological Applications (SRTA-City), Alexandria, Egypt

**Keywords:** COVID-19, epitopes, mRNA vaccine, neutralizing antibodies, RBD, spike, subunit vaccine, viral variant

## Introduction

Since the emergence of COVID-19 pandemic caused by SARS-CoV-2 in December 2019 in China and its prompt spread by March 2020 globally, the world is suffering from a vast public health crisis (1–4). As stated by the World Health Organization (WHO) and real-time data from Worldometer, the reported cases of COVID-19 exceeded 418 million cases worldwide, of which, 5.85 million deaths as of 17th February 2022 (https://www.worldometers.info/coronavirus/countries-where-coronavirus-has-spread/). Vaccines of different types, immunization procedures and routes, and from different countries and companies unexpectedly started to appear by the end of 2020 and were approved for marketing worldwide (5–8). Nevertheless, understanding the antigen epitopes in all of these vaccines is still poor.

The public debate began, which vaccine to choose, which one is more effective and with less adverse reactions, besides the new type of vaccines [messenger RNA (mRNA)-based vaccines] added more factors to this debate. Millions worldwide still do not accept receiving the COVID-19 mRNA vaccines and fear the risk of their adverse events following immunization (AEFI) especially with the unavailability of convincingly enough data for clinical experimentation of these vaccines and long-term surveillance of their safety on recipients of different medical conditions, age, and gender. Furthermore, reports indicated that even mRNA vaccines offer high short-term protection against SARS-CoV-2 (9). On the other hand, both scientific and health communities urgently suggest that mRNA vaccination is incontestably the most effective way to provide population immunity (5–8, 10) and should be spread worldwide to put an end to the pandemic and return to normal life. The next debate is about the coverage of the continuously arising and spreading SARS-CoV-2 variants.

Neutralizing antibodies with high potency are an effective line of defense in preventing and inhibiting viral infections via interrupting viral attachment to its target cell. This ability of neutralizing antibodies depends on their specific recognition of epitopes. Therefore, knowing the interaction between antibodies and epitopes provides a basis for the rational design of vaccines. After the introduction of recombinant DNA technologies, the concept of vaccination changed from immunization with inactivated intact pathogens (full spectrum of pathogen antigens) to a new concept in which isolated antigens are used for immunization. The vaccines prepared based on the last concept are designated subunit vaccines (11). Identifying an effective subunit vaccine provides the advantage of identifying the specific epitopes responsible for the protection, then immunize with the precise epitope, corresponding to an effective neutralizing antibody. Theoretically, to identify a desirable epitope, the simplest way is to isolate this epitope-binding monoclonal antibody. Then, identifying a highly potent neutralizing monoclonal antibody, and immunizing an individual with the epitope of that monoclonal antibody. Additionally, if the monoclonal antibody can significantly cross react, it can bind a variety of genetic variants of the pathogen it neutralizes, thus immunized individuals with the epitope of this antibody will have the ability to broadly cross neutralize different variants (12).

In the current article, we aimed to outline the available data regarding the epitope profile of mRNA-based vaccine recipients in comparison to subunit vaccines and naturally infected individuals. Besides, concluding if these data confirm the proposed superior efficacy of mRNA-based vaccine-induced immunity over subunit vaccines- and natural infection-induced immunity against SARS-CoV-2 variants.

## SARS-CoV-2 and Its Variants

SARS-CoV-2 and SARS-CoV share the genome structure, which is a single-stranded positive-sense (+ss) RNA genome with a length of 29.8–29.9 kb, comprising two large ORFs that encode the polyproteins, four structural protein genes that encode envelope, membrane, spike, and nucleocapsid proteins, as well as some accessary protein genes (13, 14). For SARS-CoV-2 to enter its host cells and initiate infection, the viral spike glycoprotein interacts with the angiotensin-converting enzyme 2 (ACE2) receptor on host cells and fuses with cellular membranes (15). The surface-exposed location of the spike protein not only permits it to achieve fusion with cellular membranes but also makes it a direct and major target for host immune responses (host neutralizing antibodies) (15). Thus, the spike protein or its structural domains [e.g., receptor-binding domain (RBD)] is the primary target for eliciting potent neutralizing antibodies, and design of vaccines along with antiviral therapeutics (16–19).

The SARS-CoV-2 virus carries a high mutation rate and transmission capacity when compared to other coronaviruses. Variants of SARS-CoV-2 are classified as variant being monitored (VBM) including Alpha (B.1.1.7 and Q lineages), Beta (B.1.351 and descendent lineages), Epsilon (B.1.427 and B.1.429), Eta (B.1.525), Gamma (P.1 and descendent lineages), Kappa (B.1.617.1), Iota (B.1.526), Mu (B.1.621, B.1.621.1), Zeta (P.2), and 1.617.3, variant of interest (VOI), and variant of concern (VOC) including Delta (B.1.617.2 and AY lineages) and Omicron (B.1.1.529 and BA lineages) (20–25).

Some SARS-CoV-2 variants have mutations in their spike protein, which improve the affinity between virus and the ACE2 receptor on host cells, leading to accelerated viral transmission and substantial or complete escape from neutralizing antibodies (26–28). Furthermore, single amino acid substitutions (e.g., D429A, R441A or D454A) or deletions of numerous amino acids at the RBD C-terminal or N-terminal region were reported to completely disrupt most neutralizing epitopes in spike RBD of SARS-CoV (29). In case of SARS-CoV-2, mutations at sites 473, 475, and 476 in spike RBD are already found in a small number of viral variants, and they may have the potential to decrease the binding affinity and effectiveness of neutralizing antibodies. Meanwhile, the mutations in epitope _473_YQAGSTP_479_ found in RBM are beneficial for the virus in some unrevealed way (30).

## Epitope Profiling in RBD-Based Protein Subunit Vaccine

At present, vaccine-induced protection against SARS-CoV-2 in an individual is estimated by humoral responses, for example total antibodies titers against the viral spike RBD and half-maximal neutralization titers (NT50s) using pseudotyped or live viruses (5, 6, 8, 10). Although humoral immune responses in clinical trials for various COVID-19 vaccine candidates have been described, the absence of standardized neutralization assays makes comparing different vaccine candidates a difficult task. Yang et al. (31) carried out two randomized, double-blind, placebo-controlled, phase 1 and phase 2 trials for a recombinant tandem-repeat dimeric RBD-based protein subunit vaccine (ZF2001) against SARS-CoV-2. Their study was the first to report phase 1 and phase 2 clinical data for RBD-based protein subunit COVID-19 vaccine that comprises an alum adjuvant. In phase 1, they measured vaccine safety by the occurrence of adverse events and serious adverse events. In phase 2, they evaluated vaccine safety as well as immunogenicity by the seroconversion rate and geometric mean titers [GMTs] of neutralizing antibodies against SARS-CoV-2. They found that ZF2001 is both well tolerated and immunogenic. Phase 3 clinical trials are continuing to further explore ZF2001 safety and protective efficacy (31).

Mapping the epitope profile of antibodies elicited by both vaccines and natural infection is the key step in assessing vaccine functionality and advantage of vaccine-induced immunity. Besides, this mapping can elucidate the molecular basis of humoral immune responses. Furthermore, the evolution of vaccine-induced immune escape if considered in conjunction with viral virulence will help achieve herd immunity against COVID-19. Yang et al. (32) reported protective immunity induced by a vaccine targeting SARS-CoV-2 spike protein RBD in three animal species. They stated that antibodies induced by the vaccine and those found in the sera of patients with COVID-19 share common binding epitopes. They also observed that aluminum adjuvant can further enhance the immune response induced by the vaccine, a single vaccine dose might elicit a high level of virus-neutralizing activity, the toxicology studies in the non-human primates confirmed vaccine safety, and that vaccination protected non-human primates against an *in vivo* challenge with SARS-CoV-2 (32).

Epitope profiling in RBD-based antigens of SARS-CoV-2 revealed the critical antigenic determinants, which include three immunodominant epitopes, a highly conserved epitope (_350_VYAWN_354_) located exposed on the surface of viral spike protein trimer, a variable epitope among different viral strains (_473_YQAGSTP_479_) found in the receptor binding motif (RBM), and a highly conserved cryptic cross-reactive epitope (_407_VRQIAP_412_) shared between RBD of SARS-CoV-2 and SARS-CoV (30). These data can elucidate the humoral immune responses to the viral spike protein RBD and may enable design of new anti-SARS-CoV-2 vaccines.

## Epitope Profiling in COVID-19 mRNA Vaccines

With the appearance of SARS-CoV-2 variants that harbor mutations in key epitopes, the risk of eroding adaptive immunity elicited by either vaccination or prior infection as a result of this antigenic evolution increased. Since the breadth of epitopes targeted by vaccine-induced antibodies or natural infection-induced antibodies partially contributes to susceptibility to erosion by viral evolution, the specificity of polyclonal antibodies induced by natural infection or Moderna mRNA-1273 COVID-19 vaccine (two doses) was compared by deep mutational scanning (33). The results showed that vaccine-elicited antibodies have more targeted neutralizing activity to SARS-CoV-2 spike protein RBD than natural infection-elicited antibodies. Additionally, vaccine-elicited antibodies demonstrated greater binding breadth across multiple RBD epitopes compared to infection-elicited antibodies. Thus, single mutations in RBD seemed to have less impact on antibody immunity acquired by Moderna mRNA-1273 COVID-19 vaccine compared to prior infection (33).

Wisnewski et al. (34) mapped immunogenic amino acid motifs and linear epitopes of primary sequence of SARS-CoV-2 spike protein that induce IgG in recipients of Pfizer-BioNTech COVID-19 mRNA vaccine. The obtained data identified various distinctive amino acid motifs recognized by vaccine-elicited IgG, a subset of those recognized by IgG from natural infection (hospitalized COVID-19 patients), which can mimic 3-dimensional conformation (mimotopes). The identified dominant linear epitopes in the C-terminal region of the spike protein subunits include amino acids 558–569, 627–638, and 1,148–1,159 (34). These epitopes of COVID-19 mRNA vaccine are identical with those of SARS-CoV, bat coronavirus, and epitopes that trigger IgG during natural infection, but have limited homology to spike protein of non-pathogenic human coronavirus. The identified epitopes in COVID-19 mRNA vaccine may form the basis for further research of immune escape, viral variants, and design of vaccine and therapy.

Amanat et al. (35) revealed that polyclonal antibody responses after SARS-CoV-2 mRNA vaccination target three epitopes within viral spike protein; RBD, the N-terminal domain (NTD), and S2 subunit. They also highlighted that antibody responses after vaccination were comparable to or exceeded those after natural infection, mRNA vaccination induced a high rate of antibodies with no neutralizing activity, vaccination induced cross-reactive antibodies to seasonal human coronaviruses HKU1 and OC43, and that a proportion of vaccine-induced antibodies with binding ability to RBD can offer substantial protection against viral variants carrying single E484K RBD mutations.

Nitahara et al. (36) performed high-resolution linear epitope profiling of recipients of Pfizer-BioNTech COVID-19 mRNA vaccine and COVID-19 patients and found that vaccine-induced antibodies targeting viral spike RBD have a broader distribution across RBD than natural infection-induced antibodies. Moreover, mutation panel assays targeting the viral variants of concern demonstrated that the epitope variety induced by mRNA vaccine is rich in breadth, thus can grant resistance against viral evolutionary escapes in future, which represents an advantage of vaccine-induced immunity.

Collectively, these studies suggested that mRNA vaccination against SARS-CoV-2 elicited antibodies targeting viral spike RBD that have a broader distribution across RBD than natural infection-induced antibodies, which seem to offer more resistance against future SARS-CoV-2 evolutionary escapes. Table 1 summarizes the identified epitopes used in mRNA and subunit COVID-19 vaccines and those in natural infection.

**Table 1:** Comparison between the identified epitopes in mRNA and subunit COVID-19 vaccines and those in natural infection.

**Subjects**	**Identified epitopes**	**Epitopes position**	**Reference**
Naturally infected individuals with disease severity of mild non-hospitalized, moderate hospitalized, and severe admitted to the intensive care unit	155 AAIVLQLPQGTTLPKGF 171	Epitope 155–171 from N protein	(37)
RBD-based antigens (subunit vaccine)	350 VYAWN 354	Exposed on the surface of viral spike protein trimer	(30)
	473 YQAGSTP 479	In the receptor binding motif	
	407 VRQIAP 412	Shared between RBD of SARS-CoV-2 and SARS-CoV	
mRNA vaccine and natural infection	Amino acids 558–569, 627–638, and 1148–1159	C-terminal region of the spike protein subunits	(34)
BNT162b2 mRNA vaccine and natural infection	N394 to A411, T415 to F429, V433 to N450, R457 to S477, recognized by both vaccine recipient and patient sera N334 to A348, S373 to L390, S514 to F541, recognized only by vaccine-elicited sera Substituting amino acids K417N, K417T, E484K, and N501Y, recognized only by vaccine-elicited sera	Within the RBD Single amino acid mutations of RBD in B.1.1.7, B.1.351, and P.1 variants	(36)

## Impact of Viral Variants on mRNA and Subunit Vaccines-Induced Immunity

BNT162b2 mRNA vaccine was reported to elicit neutralization of spike glycoproteins of B.1.617.1, B.1.617.2, B.1.618 Indian SARS-CoV-2 variants or B.1.525 (Nigeria lineage) after 2–4 weeks after second dose (38). However, neutralization titres against the variants (especially the B.1.617.1 variant) were lower than that against the original virus. In addition, the durability of neutralization titres against the variants was not examined. These variants with mutations in spike glycoproteins were reported to have the potential to change neutralization by influencing spike function instead of antigenicity, despite the fact that these variants revealed similar specific infectivity and infectious titres to the original virus. In a more recent study, it was found that neutralizing titers were reduced more than 22-fold against variant of concern Omicron (B.1.1.529) compared with Wuhan-neutralizing titers after two doses of BNT162b2 (39). One month after the third dose, neutralizing titers to Omicron were increased 23-fold in relation to those after two doses and were comparable to Wuhan-neutralizing titers after two doses. These findings suggested the need for three doses (vaccine boosters) of BNT162b2 mRNA vaccine to protect against Omicron variant.

The development of humoral and T cell responses against ancesteral SARS-CoV-2 and B.1.351, B.1.617.2, and P.1 variants that carry specific mutations in the spike gene was analyzed in previously infected (recovered) or uninfected (naive) individuals who received mRNA SARS-CoV-2 vaccines (40). Previously infected individuals sustained higher antibody titres against both the original virus and variants than uninfected ones, yet uninfected post-vaccination individuals reached equivalent neutralization against the original virus after second dose. Overall, the neutralization capacity was reduced against the variants, but B.1.351 and P.1 had the greatest reduction. These data pointed to vaccine boosters as an approach to alleviate the effect of continously emerging new SARS-CoV-2 variants with mutations in major neutralizing antibody-binding sites on the longevity of vaccine-induced immune protection. *In vitro* stimulation analysis using spike or nucleocapsid peptide revealed that T cell activation markers progressively increased after vaccination.

Extensive neutralization against Delta and Omicron variants elicited by Omicron-specific subunit vaccine booster is another example for the importance of booster doses to face the surge of variants and ensure that SARS-CoV-2 variants carrying a great number of mutations will not escape vaccine-mediated protection (41). A third dose of mRNA vaccine could provide strong (91%) protection against Delta SARS-CoV-2 variant in frontline workers (42). ZF2001 subunit vaccine booster following two doses of inactivated vaccines was found to increase the neutralizing antibody titer against SARS-CoV-2 and its variants in mice, particularly against Delta variant (43). This heterologous booster vaccine was recommended for future immunization program. Heterologous booster of recombinant protein subunit vaccine after two doses of inactivated whole-virion vaccines was evidenced to be highly immunogenic and safe for healthy adults and could significantly increase anti-RBD responses and neutralizing titers against SARS-CoV-2 and its variants comprising B.1.617.2 (Delta variant), B.1.351, B.1.1.7, and P.1 (44).

While COVID-19 vaccines are ideally designed to elicit neutralizing antibodies against viral spike protein, the mRNA vaccines approved so far provided efficient protection even after only one dose, when just non-neutralizing antibodies and moderate responses of T helper 1 cell are detectable, whereas almost no neutralizing antibodies found (45). These data recommended that vaccine-mediated protection probably needs low neutralizing antibodies levels and might involve other effectors such as T cells, non-neutralizing antibodies, and innate immune mechanisms. T cell responses prime early and contribute to protection but are comparatively impaired in severe disease leading to intense activation and lymphopenia (46). T cell memory incorporates extensive recognition of SARS-CoV-2 proteins, determined at about 30 epitopes/individual, and is well-sustained to date. This extensive recognition may limit the impact of individual viral mutations and can offer protection against severe disease caused by viral variants, even Omicron (46). The cellular immune response induced by BNT162b2 mRNA vaccine among healthcare workers in Bulgaria was investigated in the course of 16 weeks after first dose (47). One month after completing immunization, the number of virus-specific T cells producing IFNγ significantly correlated with virus-neutralizing activity and RBD-specific IgA levels induced after first dose. However, detection of T cells producing IFNγ may need longer stimulation beyond the first month after completing immunization (47).

## Final Considerations

In this opinion article, we discussed the epitope profile of COVID-19 mRNA-based vaccine recipients in comparison to subunit vaccines and naturally infected individuals and subsequent superior efficacy of mRNA-based vaccine-induced immunity against SARS-CoV-2 variants. To the best of our knowledge, there are currently no clear and sufficient published data concerning the antigen epitopes in each type of marketed COVID-19 vaccines, or available studies that compare the efficiency and functionality of inactivated vaccine-, subunit vaccine-, viral-vectored vaccine-, and mRNA vaccine-induced immunity against either SARS-CoV-2 or its variants based on epitope profile. However, some preliminary evidences point that mRNA vaccination against SARS-CoV-2 has induced antibodies targeting viral spike RBD that have a broader distribution across RBD than natural infection-induced antibodies, which seem to offer more resistance against future SARS-CoV-2 evolutionary escapes. Studies to comprehensively assess the epitope variety induced by each COVID-19 vaccination have yet to be conducted to raise public confidence in COVID-19 vaccines, promote their functionality and advantage of vaccine-induced immunity in spite of associated AEFI. This research if conducted would have tremendous impact on vaccination strategies to face the pandemic and viral variants of concern. Moreover, the method applied to comprehensively assess the epitope variety for vaccination against viral variants should be carefully considered.

## Author Contributions

NA and AA drafted, critically revised the manuscript, and agree to be accountable for the content of the work. All authors contributed to the article and approved the submitted version.

## Conflict of Interest

The authors declare that the research was conducted in the absence of any commercial or financial relationships that could be construed as a potential conflict of interest.

## Publisher's Note

All claims expressed in this article are solely those of the authors and do not necessarily represent those of their affiliated organizations, or those of the publisher, the editors and the reviewers. Any product that may be evaluated in this article, or claim that may be made by its manufacturer, is not guaranteed or endorsed by the publisher.
